# Characterization of Comments About bioRxiv and medRxiv Preprints

**DOI:** 10.1001/jamanetworkopen.2023.31410

**Published:** 2023-08-30

**Authors:** Clarissa França Dias Carneiro, Gabriel Gonçalves da Costa, Kleber Neves, Mariana Boechat Abreu, Pedro Batista Tan, Danielle Rayêe, Flávia Zacouteguy Boos, Roberta Andrejew, Tiago Lubiana, Mario Malički, Olavo Bohrer Amaral

**Affiliations:** 1 Institute of Medical Biochemistry Leopoldo de Meis, Universidade Federal do Rio de Janeiro, Rio de Janeiro, Brazil; 2 Berlin Institute of Health at Charité–Universitätsmedizin Berlin, QUEST Center for Responsible Research, Berlin, Germany; 3 Carlos Chagas Filho Institute of Biophysics, Universidade Federal do Rio de Janeiro, Rio de Janeiro, Brazil; 4 Department of Gastrointestinal Oncology, Netherlands Cancer Institute, Amsterdam, the Netherlands; 5 Division of Molecular Carcinogenesis, Netherlands Cancer Institute, Amsterdam, the Netherlands; 6 Oncode Institute, Utrecht, the Netherlands; 7 Department of Ophthalmology and Visual Sciences, Albert Einstein College of Medicine, Bronx, New York; 8 Programa de Pós-graduação em Psicobiologia, Universidade Federal de São Paulo, São Paulo, Brazil; 9 Department of Biochemistry, Institute of Chemistry, Universidade de São Paulo, São Paulo, Brazil; 10 Ronin Institute, Virtual Organization, São Paulo, Brazil; 11 School of Pharmaceutical Sciences, University of São Paulo, São Paulo, Brazil; 12 Department of Epidemiology and Population Health, Stanford University, Stanford, California; 13 Stanford Program on Research Rigor and Reproducibility, Stanford University, Stanford, California; 14 Meta-Research Innovation Center at Stanford, Stanford University, Stanford, California

## Abstract

**Importance:**

Preprints have been increasingly used in biomedical science, and a key feature of many platforms is public commenting. The content of these comments, however, has not been well studied, and it is unclear whether they resemble those found in journal peer review.

**Objective:**

To describe the content of comments on the bioRxiv and medRxiv preprint platforms.

**Design, Setting, and Participants:**

In this cross-sectional study, preprints posted on the bioRxiv and medRxiv platforms in 2020 were accessed through each platform’s application programming interface on March 29, 2021, and a random sample of preprints containing between 1 and 20 comments was evaluated independently by 3 evaluators using an instrument to assess their features and general content.

**Main Outcome and Measures:**

The numbers and percentages of comments from authors or nonauthors were assessed, and the comments from nonauthors were assessed for content. These nonauthor comments were assessed to determine whether they included compliments, criticisms, corrections, suggestions, or questions, as well as their topics (eg, relevance, interpretation, and methods). Nonauthor comments were also analyzed to determine whether they included references, provided a summary of the findings, or questioned the preprint’s conclusions.

**Results:**

Of 52 736 preprints, 3850 (7.3%) received at least 1 comment (mean [SD] follow-up, 7.5 [3.6] months), and the 1921 assessed comments (from 1037 preprints) had a median length of 43 words (range, 1-3172 words). The criticisms, corrections, or suggestions present in 694 of 1125 comments (61.7%) were the most prevalent content, followed by compliments (n = 428 [38.0%]) and questions (n = 393 [35.0%]). Criticisms usually regarded interpretation (n = 286), methodological design (n = 267), and data collection (n = 238), while compliments were mainly about relevance (n = 111) and implications (n = 72).

**Conclusions and Relevance:**

In this cross-sectional study of preprint comments, topics commonly associated with journal peer review were frequent. However, only a small percentage of preprints posted on the bioRxiv and medRxiv platforms in 2020 received comments on these platforms. A clearer taxonomy of peer review roles would help to describe whether postpublication peer review fulfills them.

## Key Points

**Question:**

What is the content of the comments posted on the bioRxiv and medRxiv preprint platforms?

**Findings:**

In this cross-sectional study, 7.3% of preprints from 2020 had received at least 1 comment (mean follow-up of 7.5 months), with a median length of 43 words. Criticisms, corrections, or suggestions (most commonly regarding interpretation, methodological design, and data collection) were the most prevalent types of content in these comments, followed by compliments and questions.

**Meaning:**

This study found that, although rare, when comments were present on the preprint platforms, they addressed relevant topics that would be expected to emerge from peer review.

## Teaser

This cross-sectional study assesses the content of comments posted on the bioRxiv and medRxiv platforms in 2020, using a predefined taxonomy based on qualitative studies of peer review.

## Introduction

Preprints have recently gained attention from the biomedical science community,^1^ with their adoption increasing since the 2010s^2^ and growing rapidly after the COVID-19 pandemic.^3^ A frequently mentioned advantage of preprints is the possibility of feedback.^4^ Nevertheless, engagement with postpublication peer review remains underwhelming,^5^ with less than 10% of bioRxiv and medRxiv preprints receiving comments.^6,7^

After the COVID-19 pandemic, however, increased attention was directed at preprints, and comments were present in 16% of a 2020 sample of COVID-related preprints.^3^ Nevertheless, their content has not been studied in detail, to our knowledge; thus, it remains unclear whether they resemble the feedback provided by journal-elicited peer review or not. Such an assessment could inform future discussions on publication models involving preprints and postpublication peer review.^8^ In the present cross-sectional study, we describe the content of comments posted on the bioRxiv and medRxiv platforms in 2020, using a predefined taxonomy based on qualitative studies of peer review.

## Methods

A protocol was registered prior to data collection at the Open Science Framework (OSF) website.^9^ Deviations^10^ are summarized and code and data^11^ are also available at the OSF website. Institutional review board approval is not applicable to this study because it does not involve human research.^12^ This cross-sectional study followed the Strengthening the Reporting of Observational Studies in Epidemiology (STROBE) reporting guideline.^13^

### Study Sample

All preprints posted on the bioRxiv and medRxiv platforms in 2020 were accessed through each platform’s application programming interface (API) on March 29, 2021 (ie, between 2 and 13 months after date of posting; mean [SD], 7.5 [3.6] months). The platforms were selected based on their use, relevance, and comment accessibility. We selected 1903 preprints from bioRxiv and 1108 from medRxiv with at least 1 comment to their first version. Those with more than 20 comments (5 preprints from bioRxiv and 29 preprints from medRxiv) were excluded to avoid oversampling particular preprints, and the remaining preprints were randomly sampled until 1000 comments were reached for each platform. Of these preprints, 79 were not assessed due to discrepancies between API data and online availability, leaving a total of 1921 preprints. Because this was an exploratory study with no a priori statistical hypotheses, sample size was determined based on feasibility.

### Analysis of Comments

A data collection form was built based on studies about peer review content^14^ and preprint comments.^6^ An instruction manual detailing the content classification scheme was made available to comment evaluators (including C.F.D.C, G.G.d.C., K.N., M.B.A., P.B.T., D.R., F.Z.B., R.A, T.L., and M.M.).^15^

Evaluators initially participated in a pilot study to refine our taxonomy, including a sample of 17 comments. After consolidation of the data collection form, they received a training set of 12 comments consensually evaluated by the coordinating team (including C.F.D.C, G.G.d.C., K.N., and M.B.A.). Consensus answers were visible after evaluators completed their assessments, to allow resolution of queries and discrepancies. These comments were not included in the final sample.

Each sampled preprint was assigned to 3 evaluators, who filled out the data collection form (between April and September 2021) for all comments that the preprint received before March 29, 2021. Agreement was measured by Fleiss κ coefficients among all evaluators for each question and by the percentage of agreement for all questions for each evaluator pair.

**Figure 1.**
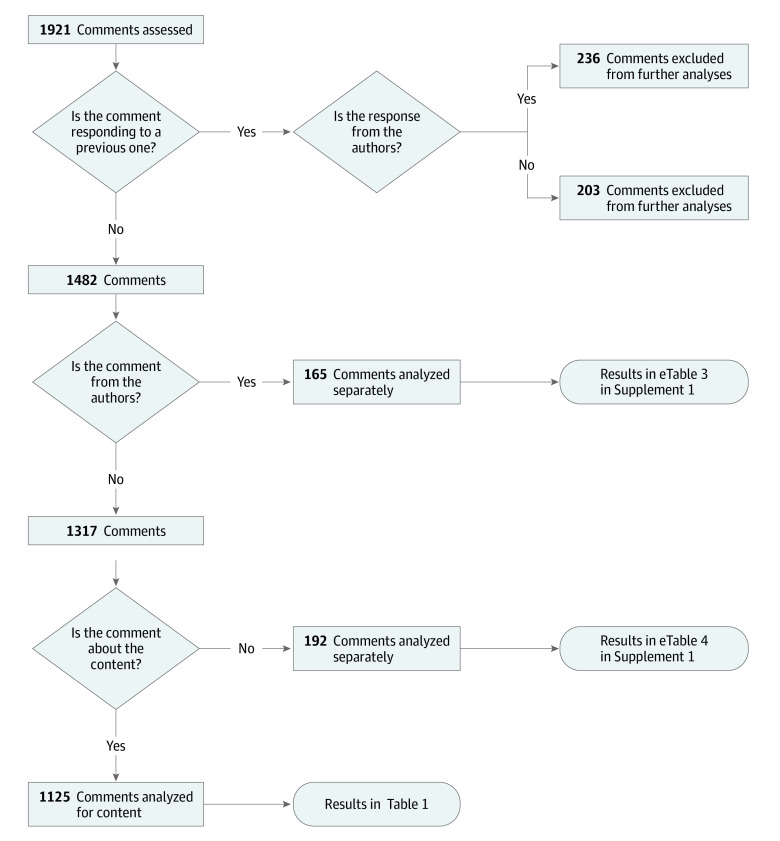
Flow Diagram of the Analysis Process Responses to other comments were not analyzed. Comments from the preprint authors and those classified as not about the preprint content were analyzed using specific categories. All other comments (ie, not responses, from nonauthors, and about the content) were analyzed using the main data extraction form.

Figure 1 summarizes the process of comment coding. Evaluators first identified whether comments were responses to a previous comment, whether they were made by one of the preprint’s authors, and whether they referred to the preprint’s content. Responses did not have their content analyzed.

Comments from preprint authors were classified according to their entire content using a preset list of nonexclusive categories^15^ (adapted from Malički et al^6^). Evaluators could also choose “other” and provide a description. After reviewing these descriptions, we reclassified them under existing categories or under a newly created one (extra materials).

When a nonauthor comment was classified as not about the content, the evaluator was asked to describe it. Descriptions yielded the classification presented in eTable 4 in Supplement 1. All other comments were evaluated using the main data collection form.

Concerning format, we asked whether nonauthor comments were clearly written (as subjectively assessed by evaluators), included personal offenses (to the authors or in general), or were products of organized review efforts. We also asked whether they contained a summary description of the preprint, new data or analyses, the types of references included (if any), and whether they questioned the preprint’s conclusions.

General content was assessed by asking whether the comments included (1) criticisms, corrections, or suggestions; (2) compliments; (3) questions; or (4) other content not fitting these categories. Criticisms, corrections, or suggestions; compliments; questions; and other general content were further evaluated concerning their specific content categories.^15^ Evaluators could also select “other” and provide a description. No new categories were identified, and comments were reclassified under existing categories.

For questions with mutually exclusive categories, the most prevalent answer was considered final. In case of triple disagreements, a fourth evaluator reviewed the questions and made the final decision (deviating from our original protocol). For questions in which categories were not mutually exclusive, agreement was not required, and every option selected by at least 1 evaluator was considered.

### Additional Variables

For each comment, we collected the number of words, counting hyperlinks as single words. In addition to the protocol, we classified the type of organized review effort when present. For each preprint, we collected the date of posting, subject area (according to the platform), region of corresponding author’s affiliation, and whether it had been published in a journal (data collected on October 27, 2021) or withdrawn (data collected on December 1-14, 2021). If published, we collected the publication date and venue with its 2020 Journal Citation Report impact factor. For both preprints and published versions, we collected Altmetric Attention Scores^16^ (the score can be [potentially] increased unlimitedly; the higher the value, the more attention a preprint received) and citation counts (on October 27 and 28, 2021, respectively). Preprints were classified as related to COVID-19 by searching title and abstract for “coronavirus,” “covid-19,” “sars-cov,” “ncov-2019,” “2019-ncov,” “hcov-19,” and “sars-2.”^3^

### Statistical Analysis

For each question, we present the frequency of “yes” answers. Proportions are relative to the number of comments eligible for assessment in each question, excluding those removed in previous data collection steps (Figure 1). For general content and format, 95% CIs for proportions are included. In addition to the protocol, we present the frequency of “yes” answers in subsets of comments (those from organized review efforts, those questioning the preprint conclusions, and those that received a response).

We also included additional analyses of general content and format using the preprint rather than the comment as the unit of analysis. For this, we counted the preprints in which at least 1 comment presented a feature in relation to the total applicable preprints in each question, following the exclusion flow (Figure 1).

To explore associations between comment content and preprint features, we used logistic regressions in which each content category was taken as the response variable (using “no” as the reference), presenting *P* values from analysis of deviance tests. We do not label any *P* value as significant or not because this is an exploratory study, and false-positive rates are likely inflated due to the multiple comparisons tested. Analyses were restricted to general questions in which each response category had a prevalence of at least 5%. Analyses were performed in R, version 4.2.2 (R Project for Statistical Computing).^17^ All analyses were performed between September 2021 and August 2022 and last reviewed in July 2023.

## Results

### Sample Description

**Figure 2.**
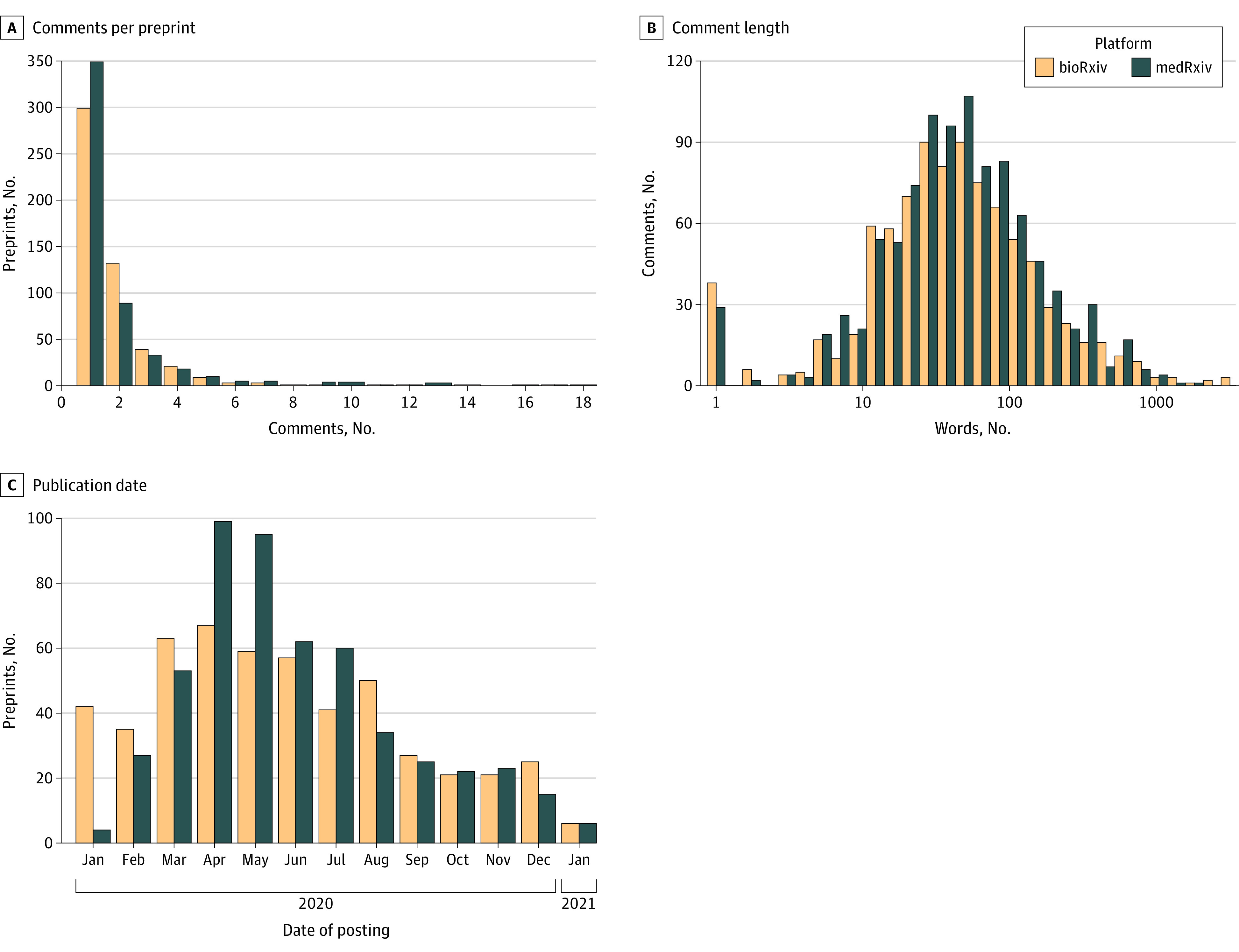
Sample Description Bars represent the total number of preprints in each bin (ie, those from bioRxiv and those from medRxiv). A, Number of comments per sampled preprint. The median is 1, with a maximum of 17 for bioRxiv and 18 for medRxiv (those with >20 comments were excluded). B, Distribution of comment length. The median length is 42 words (range, 1-3172 words) on the bioRxiv platform and 44 words (range, 1-1640 words) on the medRxiv platform; the overall median length is 43 words (range, 1-1640 words). The peak in 1-word comments mostly consists of isolated hyperlinks. C, Distribution of number of comments by publication month.

We identified 52 736 preprints from 2020, 7.3% (n = 3850) of which had comments. Considering only first versions with fewer than 20 comments, there were 3070 comments from 1898 bioRxiv preprints and 2316 comments from 1079 medRxiv preprints for sampling. Data collection was completed for 1921 comments from 1037 preprints. Figure 2 shows the number of comments per preprint, the comments’ lengths, and the preprint publication dates.

Preprints in our sample had a median of 3 citations (IQR, 1-12 citations), a median Altmetric Attention Score of 21.2 (IQR, 5.5-119.4), and most were from authors based in North America and Europe; 91.4% of medRxiv preprints (480 of 525) and 35.0% of bioRxiv preprints (176 of 508) were related to the COVID-19 pandemic, which was included in the most common categories in each platform (eFigure 1 in Supplement 1); 29.0% of the preprints (300 of 1033) had been published in a peer-reviewed journal (mean [SD] impact factor of 11.1 [12.9]) by October 27, 2021, and 4 of them were later withdrawn.

### Content of Comments

Agreement between evaluators is presented in eTables 1 and 2 in Supplement 1. Most disagreements occurred when one of the evaluators chose different answers at the initial stages of the form (eg, whether comments were about the preprint’s content), resulting in disagreement on subsequent categories as well.

The evaluation flow is described in Figure 1; 439 comments were identified as responses and excluded from further analyses. Of the 1482 remaining comments, 165 (11.1%) were posted by preprint authors. Most commonly, these were updates to the preprint’s publication status (89 of 165 [54.0%]), additional information on the study (49 of 165 [30.0%]), or corrections (29 of 165 [18.0%]) (eTable 3 in Supplement 1). The remaining 1317 comments were from nonauthors, of which 192 comments (14.6%) were not about the preprint content (eTable 4 in Supplement 1). Notably, 33.3% (n = 64) were links to other review or commenting platforms (61 from Oxford Immunology Network COVID-19 Literature Reviews and 1 each from PreReview, Publons, and DataMethods).

Of the 1125 comments about the preprint content, 694 (61.7%) included a criticism, correction, or suggestion, while 428 (38.0%) included compliments or positive appraisals and 393 (35.0%) included questions; 6.1% (69 of 1125) of comments addressed specific content but were not classified under these categories (Table). The overlap of these categories is shown in eFigure 2 in Supplement 1. Most compliments (233 of 428 [54.4%]) were present alongside criticisms, corrections, or suggestions, but only 34.0% (233 of 694) of the comments making criticisms, corrections, or suggestions included a compliment. They were mostly assertions about the interpretation or implications of the results, with no positive or negative tone.

**Table.** General Content and Format of Comments^a^

General content	No. (%) [95% CI]
Includes a criticism, correction, or suggestion	694 (61.7) [58.8 to 64.5]
Includes a compliment or positive appraisal	428 (38.0) [35.2 to 40.9]
Includes a question	393 (34.9) [32.1 to 37.7]
Includes other content (ie, not a criticism, correction, suggestion, compliment, or question)	69 (6.1) [4.7 to 7.5]
Includes references	284 (25.2) [22.7 to 27.8]
Includes a summary description	110 (9.8) [8.0 to 11.5]
Explicitly questions a conclusion of the article	98 (8.7) [7.1 to 10.4]
Provides new data	12 (1.1) [0.5 to 1.7]
Provides new analyses	5 (0.4) [0.1 to 0.8]
Provides both new data and analyses	4 (0.4) [0.0 to 0.7]
Presented clearly enough for understanding	1109 (98.6) [97.9 to 99.3]
From an organized review effort	75 (6.7) [5.2 to 8.1]
Offensive (to the authors)	2 (0.2) [0 to 0.4]

^a^ The content categories are not mutually exclusive; thus, the sum of percentages is greater than 100%. These categories are applicable to comments from nonauthors and about the content of the preprint (1125 comments).

Of the 1125 assessed comments, 110 (9.8%) included a summary description of the preprint’s findings, and a similar proportion (98 of 1125 [8.7%]) openly questioned its conclusions. On the other hand, very few comments included additional data or analyses. References were found in 25.2% (284 of 1125) of the assessed comments, and most (186 of 284 [65.5%]) included journal articles, but many other types were identified (eTable 5 in Supplement 1).

Most comments presented their points clearly (1109 of 1125 [98.6%]), and only 2 (0.2%) were identified as offensive, probably reflecting moderation policies instituted by both platforms. We identified 75 of 1125 comments (6.7%) as products of organized review efforts, of which 54 (72.0%) were from a single initiative (Sinai Immunology Review Project). Other types of efforts included journal clubs, automated screening tools, and graduate-level classes (eTable 6 in Supplement 1).

**Figure 3.**
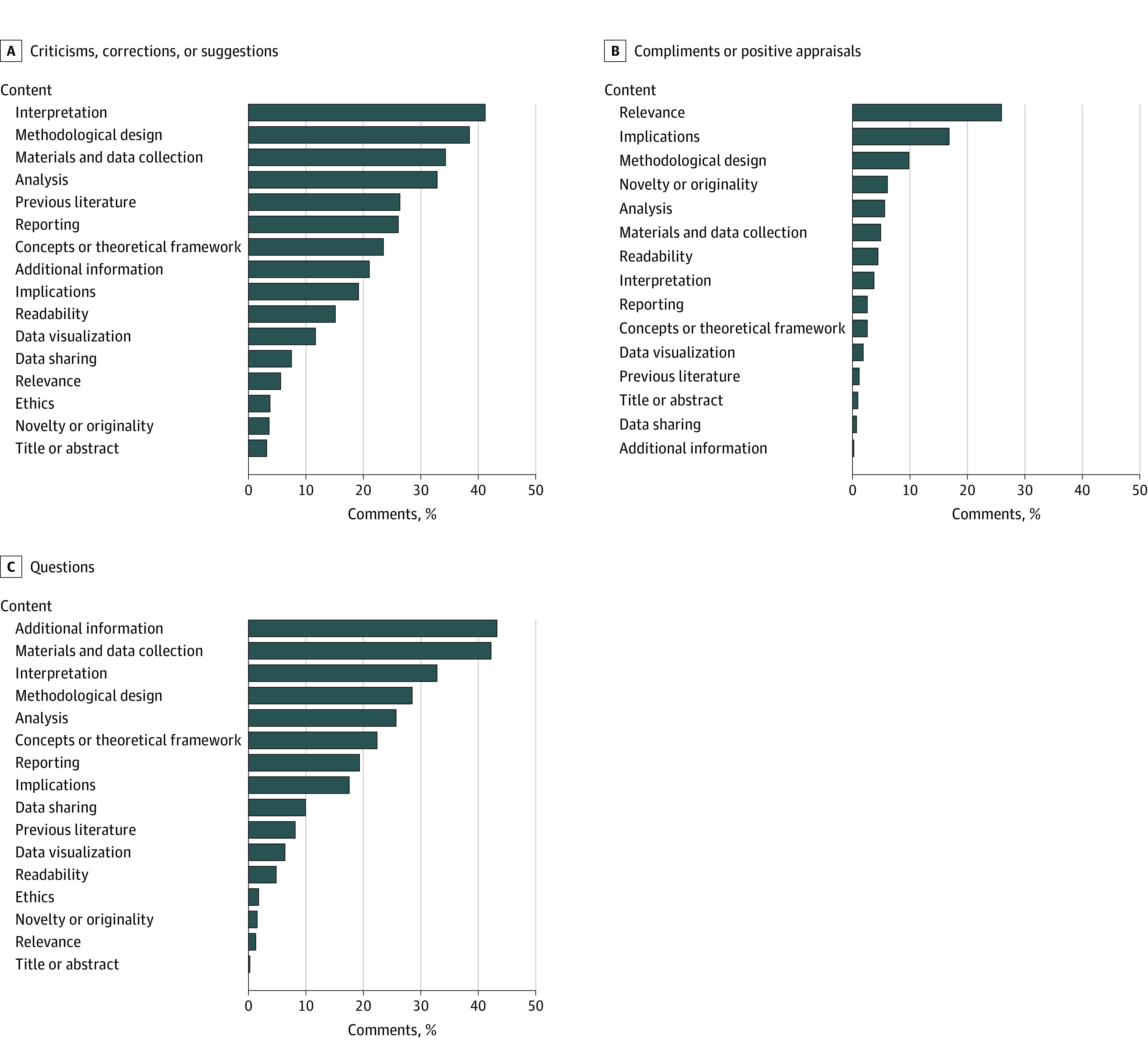
Specific Content of Comments A, Specific content of criticisms, corrections, or suggestions. B, Specific content of compliments. C, Specific content of questions. Categories are not mutually exclusive (ie, each comment could include multiple categories); thus, percentages do not add up to 100%.

Concerning specific content, criticisms, corrections, or suggestions most commonly addressed the interpretation of results (n = 286) and methods, including methodological design (n = 267), data collection (n = 238), and analysis (n = 228) (Figure 3A). Only 1 comment was a general criticism (ie, not directed at any specific aspect of the preprint); in contrast, most compliments or positive appraisals (279 of 428 [65.2%]) were general. When compliments addressed specific points, they were mostly about relevance (n = 111) and potential implications (n = 72) (Figure 3B). Questions mostly asked about information not present in the manuscript (n = 170), including other results, analyses, or visualizations, followed by inquiries about materials and data collection (n = 166) (Figure 3C). eFigure 3A in Supplement 1 presents the aggregate results of all subcategories, and selected examples are shown in eTable 7 in Supplement 1.

General and specific content categories were also analyzed by aggregating all comments from each preprint. Across 810 preprints with at least 1 comment from nonauthors about their content, distributions of content types (eTable 8 in Supplement 1) and specific categories addressed (eFigure 3B in Supplement 1) were largely similar to those at the comment level. The number of comments per preprint was moderately correlated with the percentage of specific content categories covered by their aggregate (*r* = 0.37) (eFigure 4 in Supplement 1).

### Subset Analyses

Distinctive features were observed in specific subsets of comments (eTable 9 in Supplement 1). Those comments from organized review efforts were longer (median length, 426 words [range, 45-3172 words]) and included compliments and criticisms, corrections, or suggestions more often than the overall sample of comments (52.0% [39 of 75] vs 38.0% [428 of 1125] included compliments and 99.0% [74 of 75] vs 61.7% [694 of 1125] included criticisms, corrections, or suggestions). They also included summary descriptions and references much more frequently (88.0% [66 of 75] vs 9.8% [110 of 1125] included summary descriptions and 44.0% [33 of 75] vs 25.2% [284 of 1125] included references). Comments that questioned the preprint’s conclusions were more critical (95.0% [93 of 98] vs 61.7% [694 of 1125] included criticisms, corrections, or suggestions) and less positive (5.1% [5 of 98] vs 38.0% [428 of 1125] included compliments). A larger proportion of them included references (32.0% [31 of 98] vs 25.2% [284 of 1125]) and provided new data (7.1% [7 of 98] vs 1.1% [12 of 1125]). Comments that elicited a response within the analyzed period did not differ much from the complete sample, except in the proportion of comments with questions (43.5% [87 of 200] and 44.0% [40 of 91] among comments with author and nonauthor responses, respectively, vs 35.0% [393 of 1125]).

### Associations With Preprint Features

Finally, we studied associations between the content of comments and preprint features (eTables 10 and 11 in Supplement 1). These were mostly weak, except for those of longer comments with the presence of a summary description (mean length, 539 vs 84 words; *P* = 1.6 × 10^−46^) and origin from an organized review effort (mean length, 632 vs 93 words; *P* = 2.5 × 10^−33^). Longer comments were also associated with the presence of references (mean length, 241 vs 89 words; *P* = 2.9 × 10^−17^) and of criticisms, corrections, or suggestions (mean length, 173 vs 52 words; *P* = 1.2 × 10^−35^). Preprints with comments from authors had lower Altmetric Attention Scores and a lower number of citations than the rest of our sample (mean score, 90 vs 568; *P* = 2.2 × 10^−14^; mean number of citations, 24 vs 177 citations; *P* = 1.1 × 10^−5^, respectively).

### COVID-19–Related Content

The year 2020 is remarkable for the number of preprints addressing COVID-19, which represent 63.5% of our sample (656 of 1033). Preprints with COVID-19–related content had fewer comments from authors (8.4% [86 of 1019] vs 17.2% [79 of 459]; *P* = 1.6 × 10^−6^) and fewer with compliments (29.4% [227 of 771] vs 56.6% [198 of 350]; *P* = 7.1 × 10^−18^) and more comments questioning their conclusions (10.8% [83 of 770] vs 4.3% [15 of 350]; *P* = 1.5 × 10^−4^). They also had a higher percentage of comments not addressing their content (17.4% [162 of 933]vs 7.9% [30 of 380]; *P* = 3.6 × 10^−6^) or without references (79.4% [612 of 771] vs 64.6% [226 of 350]; *P* = 2.1 × 10^−7^), suggesting that some of these might be outside standard academic discourse.

## Discussion

In this study, we analyzed the content of 1482 comments posted on 1026 preprints from bioRxiv and medRxiv; 165 (11.1%) of these comments were made by authors, most commonly providing updates about publication status. A study^6^ analyzing bioRxiv preprints with a single comment before the COVID-19 pandemic found a higher proportion of author comments (31.1%) but a similar distribution of content, with focus on publication status and additional information.

Of the remaining comments, 85.4% (1125 of 1317) were about the content of the preprint; of these, 61.7% (694 of 1125) included criticisms, corrections, or suggestions, mainly about interpretation and methods. The high proportion of specific criticisms, corrections, or suggestions, as well as questions, highlights the potential of commenting to improve preprints. However, we did not assess changes in subsequent versions to check if these comments led to changes.

While some might question whether preprint comments could fulfill the roles usually attributed to peer review, the task of the commenter seems different from that of reviewers. On average, comments are much shorter than peer-reviewed reports (a mean [SD] of 99.3 [213.0] words, much smaller than the reported mean of 477 words for open journal peer review^18^). On the other hand, comments on preprints may be more cordial; a study on peer-reviewed reports found that 7% to 10% include comments demeaning or attacking the authors,^19^ which were almost absent from our sample, likely due to the moderation provided by preprint platforms, which may be stricter than that exerted by journal editors.

Comments in our sample generally focused on domains similar to those expected from peer review, such as assessing relevance, novelty, methods, and interpretation,^14,20^ although this could be a consequence of our taxonomy being inspired by a review of peer reviewer roles.^14^ When providing criticisms, corrections, or suggestions, comments most often addressed interpretation, study design, data collection, and analysis. Comments on relevance or novelty were mostly presented as compliments. It was uncommon, however, for individual comments to address all of these aspects, and our analysis at the preprint level suggests that this was not fulfilled by their aggregate either. Meanwhile, the lack of an established taxonomy for peer review hinders further studies that aim to compare prepublication and postpublication reviews directly and more broadly.

Of 1317 nonauthor comments, 192 (14.6%) were not about the content of the preprint. Approximately one-third of these, however, were links to other peer review platforms and would probably be about the preprint had we assessed external content. Another one-third addressed the topic under study more broadly, such as questions related to health advice. This brings up the question of who are the commenters; in many cases, they were clearly someone from the same scientific field, while in other cases, they appeared to come from outside academia. However, making this distinction accurately was not always possible.

Finally, any discussion of the systemic role of comments as a form of postpublication peer review must consider the fact that only 7.3% of the preprints in our sample received comments on the preprint platforms. This is less than previously reported for COVID-19 preprints^3^ but similar to other general samples.^6,7^ Still, even the estimate for COVID-19 preprints at a time of peak attention (16%) indicates that commenting on preprints is rare—although probably more common than postpublication comments on journal articles.

### Limitations

This study has some limitations. Our findings are not necessarily generalizable to other periods in time. The year 2020 was chosen due to the increase in the number of comments,^3^ but it was also an atypical year; most of our sample was COVID-19 related, and comments in these preprints show some distinctive features, such as more frequent questioning of study conclusions. New studies would be needed to assess whether such features still hold after the end of the pandemic.

In addition, although we built our taxonomy based on academic peer review, a nonnegligible portion of comments seemed to be an extension of polarized opinions on COVID-19 expressed in social media. Nevertheless, many of these comments still fit our categories when taken literally, which may have led us to overestimate their relevance.

Our sampling strategy deliberately excluded preprints with more than 20 comments. Although this helped us prevent oversampling, our findings cannot be generalized to these heavily discussed preprints, and further studies would be needed to assess the content of comments in these particular (and infrequent) cases.

## Conclusions

This cross-sectional study found that, despite being an underused mechanism for providing feedback, comments posted on the bioRxiv and medRxiv preprint platforms have features that resemble traditional forms of peer review. Most comments were critical and focused on the interpretation of results and methodological aspects. References, particularly to peer-reviewed publications, are present in approximatley one-third of them, suggesting an academic debate is taking place. Nevertheless, these comments exist for a minority of preprints, and the extent to which other postpublication review forums or social media might be filling this gap is unclear.

Our assessment portrays a culture in transition in terms of preprint adoption and postpublication peer review. It also describes a moment of global health emergency, leading to features that may not persist. In the meantime, aggregating and organizing comments, reviews, and feedback from multiple sources to build a more robust postpublication peer review record, as well as developing a taxonomy for classifying their content, should be regarded as priorities for improving scholarly communication.

## Notes

Supplement 1. **eTable 1.** Fleiss’ Kappa Between the 3 Evaluators for Different Questions in the Form **eTable 2.** Agreement Levels Across Evaluator Pairs **eTable 3.** Specific Content Categories of Comments Posted by One of the Preprint’s Authors **eTable 4.** Comments Classified as Not Addressing the Content of the Preprint **eTable 5.** Types of References Cited in Comments **eTable 6.** Types of Organized Review Efforts **eTable 7.** Counts and Examples of Specific Content of Comments, Ordered by Total Number in Each Category **eTable 8.** Analysis of Comment Content Using the Preprint as the Unit of Analysis **eTable 9.** Analysis of Selected Subsets **eTable 10.** Exploratory Associations With Categorical Preprint Features **eTable 11.** Exploratory Associations With Continuous Preprint Features **eFigure 1.** Preprint Features **eFigure 2.** Venn Diagram Representing the Overlap Between the Main Content Categories Within Comments **eFigure 3.** Specific Content in All Main Categories, Including Criticisms/Corrections/Suggestions, Compliments, Questions and Comments Not Classified in Any of These Categories (Other) **eFigure 4.** Correlation Between the Number of Comments Per Preprint and the Percentage of Specific Categories Addressed by the Aggregate of All Comments

Supplement 2.
Data Sharing Statement

## References

[1] Berg JM, Bhalla N, Bourne PE, . Preprints for the life sciences. Science. 2016;352(6288):899-901. doi:10.1126/science.aaf9133 27199406

[2] Biology preprints over time. ASAPbio. Accessed March 3, 2023. https://asapbio.org/preprint-info/biology-preprints-over-time

[3] Fraser N, Brierley L, Dey G, . The evolving role of preprints in the dissemination of COVID-19 research and their impact on the science communication landscape. PLoS Biol. 2021;19(4):e3000959. doi:10.1371/journal.pbio.3000959 33798194 PMC8046348

[4] Sever R, Roeder T, Hindle S, . bioRxiv: the preprint server for biology. *bioRxiv*. Preprint posted online November 6, 2019. doi:10.1101/833400

[5] Dolgin E. PubMed Commons closes its doors to comments. Nature. February 2, 2018. doi:10.1038/d41586-018-01591-4

[6] Malički M, Costello J, Alperin JP, Maggio LA. Analysis of single comments left for bioRxiv preprints till September 2019. Biochem Med (Zagreb). 2021;31(2):020201. doi:10.11613/BM.2021.020201 33927548 PMC8047782

[7] Ross JS, Sever R, Bloom T, . medRxiv preprint submissions, posts, and key metrics, 2019-2021. In: International Congress on Peer Review and Scientific Publication. Accessed March 3, 2023. https://peerreviewcongress.org/abstract/medrxiv-preprint-submissions-posts-and-key-metrics-2019-2021/

[8] Stern BM, O’Shea EK; PLOS Biology Staff. Correction: a proposal for the future of scientific publishing in the life sciences. PLoS Biol. 2019;17(3):e3000179. doi:10.1371/journal.pbio.3000179 30840613 PMC6402637

[9] Carneiro CF, Neves K, Costa G, Abreu M, Amaral OB. Content of commentaries on biomedical sciences preprints - protocol. Open Science Framework (OSF). Center for Open Science. Accessed July 26, 2023. https://osf.io/54xwy

[10] Deviations from the protocol. Open Science Framework (OSF). Center for Open Science. Accessed July 26, 2023. https://osf.io/b6up2

[11] Content of comments on biomedical sciences preprints. Open Science Framework (OSF). Center for Open Science. Accessed July 26, 2023. https://osf.io/k9e8c/

[12] RESOLUÇÃO Nº 466, DE 12 DE DEZEMBRO DE 2012. Accessed August 10, 2023. https://conselho.saude.gov.br/resolucoes/2012/Reso466.pdf

[13] von Elm E, Altman DG, Egger M, Pocock SJ, Gøtzsche PC, Vandenbroucke JP; STROBE Initiative. Strengthening the Reporting of Observational Studies in Epidemiology (STROBE) statement: guidelines for reporting observational studies. BMJ. 2007;335(7624):806-808. doi:10.1136/bmj.39335.541782.AD 17947786 PMC2034723

[14] Glonti K, Cauchi D, Cobo E, Boutron I, Moher D, Hren D. A scoping review on the roles and tasks of peer reviewers in the manuscript review process in biomedical journals. BMC Med. 2019;17(1):118. doi:10.1186/s12916-019-1347-0 31217033 PMC6585141

[15] Detailed description of content data collection form and instructions manual. Open Science Framework (OSF). Center for Open Science. Accessed July 26, 2023. https://osf.io/rmjz3

[16] The donut and Altmetric Attention Score. Altmetric. Accessed August 1, 2023. https://www.altmetric.com/about-us/our-data/donut-and-altmetric-attention-score/

[17] R Core Team. R: A Language and Environment for Statistical Computing. Accessed July 26, 2023. https://www.R-project.org/

[18] Report: global state of peer review. Clarivate. Accessed March 3, 2023. https://clarivate.com/lp/global-state-of-peer-review-report/

[19] Gerwing TG, Allen Gerwing AM, Avery-Gomm S, Choi CY, Clements JC, Rash JA. Quantifying professionalism in peer review. Res Integr Peer Rev. 2020;5(1):9. doi:10.1186/s41073-020-00096-x 32760597 PMC7379804

[20] Superchi C, González JA, Solà I, Cobo E, Hren D, Boutron I. Tools used to assess the quality of peer review reports: a methodological systematic review. BMC Med Res Methodol. 2019;19(1):48. doi:10.1186/s12874-019-0688-x 30841850 PMC6402095

