# Supplementary material for: Characterization of Comments About bioRxiv and medRxiv Preprints
Source: JAMA Netw Open. 2023 Aug 30;6(8):e2331410. doi: 10.1001/jamanetworkopen.2023.31410 (PMC10469270; doi:10.1001/jamanetworkopen.2023.31410)
Supplement: Supplement 1. — eTable 1. Fleiss’ Kappa Between the 3 Evaluators for Different Questions in the Form eTable 2. Agreement Levels Across Evaluator Pairs eTable 3. Specific Content Categories of Comments Posted by One of the Preprint’s Authors eTable 4. Comments Classified as Not Addressing the Content of the Preprint eTable 5. Types of References Cited in Comments eTable 6. Types of Organized Review Efforts eTable 7. Counts and Examples of Specific Content of Comments, Ordered by Total Number in Each Category eTable 8. Analysis of Comment Content Using the Preprint as the Unit of Analysis eTable 9. Analysis of Selected Subsets eTable 10. Exploratory Associations With Categorical Preprint Features eTable 11. Exploratory Associations With Continuous Preprint Features eFigure 1. Preprint Features eFigure 2. Venn Diagram Representing the Overlap Between the Main Content Categories Within Comments eFigure 3. Specific Content in All Main Categories, Including Criticisms/Corrections/Suggestions, Compliments, Questions and Comments Not Classified in Any of These Categories (Other) eFigure 4. Correlation Between the Number of Comments Per Preprint and the Percentage of Specific Categories Addressed by the Aggregate of All Comments [file jamanetwopen-e2331410-s001.pdf]

Supplemental Online Content

Carneiro CFD, da Costa G, Neves K, et al. Characterization of comments about bioRxiv and medRxiv preprints. *JAMA Netw Open*. 2023;6(8):e2331410. doi:10.1001/jamanetworkopen.2023.31410

eTable 1. Fleiss' Kappa Between the 3 Evaluators for Different Questions in the Form

eTable 2. Agreement Levels Across Evaluator Pairs

eTable 3. Specific Content Categories of Comments Posted by One of the Preprint's Authors

eTable 4. Comments Classified as Not Addressing the Content of the Preprint

eTable 5. Types of References Cited in Comments

eTable 6. Types of Organized Review Efforts

eTable 7. Counts and Examples of Specific Content of Comments, Ordered by Total Number in Each Category

eTable 8. Analysis of Comment Content Using the Preprint as the Unit of Analysis

eTable 9. Analysis of Selected Subsets

eTable 10. Exploratory Associations With Categorical Preprint Features

eTable 11. Exploratory Associations With Continuous Preprint Features

eFigure 1. Preprint Features

eFigure 2. Venn Diagram Representing the Overlap Between the Main Content Categories Within Comments

© 2023 Carneiro CFD et al. *JAMA Network Open.*

eFigure 3. Specific Content in All Main Categories, Including Criticisms/Corrections/Suggestions, Compliments, Questions and Comments Not Classified in Any of These Categories (Other)

eFigure 4. Correlation Between the Number of Comments Per Preprint and the Percentage of Specific Categories Addressed by the Aggregate of All Comments

This supplemental material has been provided by the authors to give readers additional information about their work.

© 2023 Carneiro CFD et al. *JAMA Network Open.*

eTable 1 - Fleiss' kappa between the 3 evaluators for different questions in the form. Kappa values below 0.2 refer to questions with very low prevalence of one of the answers and a degree of subjectiveness, in which convergence among all 3 evaluators on infrequent answers was unlikely.

**Question**	**kappa**	**Number of comments**
Is the comment from one of the article's authors?	0.95	1922
Is the (author's) comment a response?	0.98	372
Is the (nonauthor's) comment a response?	0.97	1503
Is the comment about the content of the article?	0.48	1302
Is the comment from an organized review effort?	0.84	960
Does the comment include a summary description?	0.72	955
Is the comment constructed well enough?	0.09	944
Does the comment explicitly question any conclusion of the article?	0.39	952
Does the comment provide new data or analyses?	0.15	957
Does the comment include references?	0.86	960
Is the comment offensive?	0.08	960
Does the comment include any compliments or positive appraisals?	0.69	960
Does the comment include any criticisms, corrections or suggestions?	0.63	959
Does the comment include any questions?	0.80	959
Does the comment include any other specific content that was not classified as compliment, criticism, correction, suggestion or question?	0.11	960

© 2023 Carneiro CFD et al. *JAMA Network Open.*

eTable 2 - Agreement levels across evaluator pairs. Numbers 1 to 11 represent different evaluators. Values above the grey diagonal are the mean % agreement for all questions, while those below the grey diagonal are the number of shared evaluations (questions) for each pair. The mean agreement between all evaluator pairs was 92.4%.

	**1**	**2**	**3**	**4**	**5**	**6**	**7**	**8**	**9**	**10**	**11**	**Mean**
**1**		89.7	90.2	92.3	91.1	92.2	92.8	93.3	92.8	94.7	91.8	92.1
**2**	2082		89.8	90.4	91.1	91.1	91.4	91.8	88.6	91.3	89.4	90.5
**3**	481	368		90.6	93	91	87.9	92	96	92.1	92.6	91.5
**4**	1934	1525	866		93.7	94.2	91.8	94.7	95.7	92.5	92.5	92.8
**5**	2035	1418	567	1348		93	90.3	93.1	90.7	93.5	93	92.2
**6**	1938	1355	424	1234	1322		93.1	93.8	100	93.4	93.7	93.6
**7**	1750	1107	270	1107	891	1133		94.5	91.7	95.1	92.7	92.1
**8**	1416	1062	578	1171	1099	1057	848		92.9	93.7	93.5	93.3
**9**	157	182	84	90	133	10	126	109		93.9	90.9	93.3
**10**	1220	886	594	1069	777	1079	866	858	290		93.3	93.3
**11**	1237	929	489	1178	1135	1110	909	894	280	913		92.3

© 2023 Carneiro CFD et al. *JAMA Network Open.*

eTable 3 - Specific content categories of comments posted by one of the preprint's authors. N is the number of comments including the category, and percentages refer to the 165 comments that were classified as being from an author. Categories are not mutually exclusive (i.e. each comment might include multiple themes); thus, the sum of percentages is greater than 100%.

Category	Example	N	%
Publication status	"It has been accepted by the British Journal of Pharmacology today." (http://disq.us/p/27jtns0)	89	53.9
Additional information	"This is a reply to www.biorxiv.org/content/10...." (http://disq.us/p/27p3o51)	49	29.7
Study promotion	"(…) The tool is ideal when keeping healthcare worker safety and wellbeing perspective as priorities." (http://disq.us/p/29zk7iv)	34	20.6
Corrections	"There is an erratum between lines 146-149 (HLHF instead of HLLF in the stratification)." (http://disq.us/p/29gk782)	29	17.6
New data	"World Wildlife Fund-Mexico just announced a 2.83 ha estimate of overwinter area occupied by the eastern monarch butterfly population in Mexico this winter (…)" (http://disq.us/p/27wza05)	17	10.3
New analyses	"(…) We have retrained our model with confirmed cases by Feb. 11. We updated our prediction results. (…)" (http://disq.us/p/27ao9ez)	14	8.5
Feedback request	"(…) If you spot other mistakes, please let us know!" (http://disq.us/p/29acun8)	12	7.3
Extra materials	"Our new large population study is out! (…) https://www.medrxiv.org/con..." (http://disq.us/p/2cixi40)	11	6.7

© 2023 Carneiro CFD et al. *JAMA Network Open.*

eTable 4 – Comments classified as not addressing the content of the preprint. Categories were created based on the sample, and each comment was assigned to a single category. N is the number of comments classified in each category. Percentages refer to the 192 comments that were identified as not being about the content of the preprint.

**Category**	**Description**	**Example**	**N**	**%**
Link to external resource (peer-review)	Comments linking to other open peer-review platforms.	"I have posted a review of this on https://outbreaksci.prerevi…" (http://disq.us/p/2967lfs)	64	33.3
Topic under study	Comments about the topic directly under study or related topics, including health-care related questions.	"How long does it take infected individuals to develop detectable antibodies to SARS-CoV-2?" (preprint describes the development of a methodology) (http://disq.us/p/281wyp4)	57	29.7
Link to external resource (reference)	Comments promoting references (preprints and journal articles) without contextualization to the articles' findings.	"https://www.medrxiv.org/con... Another related papers [sic] was also published recently." (http://disq.us/p/28a1cwg)	26	13.5
Publication status	Comments with questions or updates about peer-review or publication in a journal.	"When do you expect this to be published in a journal? I'm a little picky about formatting, so I'd love to see this work in its final form." (http://disq.us/p/2a7p3ow)	8	4.2
Collaboration proposal	Comments offering materials or proposing collaborations.	"If anybody wants research or visit Rohinga Camp. Please contact (…)" (http://disq.us/p/28ua732)	7	3.6
Scholarly communication	Comments about the use of preprints, formatting, and peer-review systems.	"I really like that you've formatted it to make it readable. It seems it would only take 10-20 minutes but it makes a huge difference. There is no real reason the preprint should be in the journal submission format." (http://disq.us/p/27jklfm)	4	2.1
Interaction with stakeholders (media)	Comments about how the studies were picked up by the press or social media.	"This study was cited in a nice powerpoint (…) It is being circulated on WhatsUp [sic] (…)" (http://disq.us/p/2b90iol)	3	1.6
Link to external resource (blog)	Comments promoting blog posts without contextualizing with the articles' findings.	"An open Letter to the editor-in-chief at JAMA on the potential efficacy of Cyclosporine in COVID-19 disease: https://medium.com/…" (http://disq.us/p/29hvf3c)	3	1.6
Link to external resource (data)	Comments sharing databases without relating them to the article's findings.	"See https://www.ssrn.com/author… for three earlier relevant papers." (http://disq.us/p/29nliyl)	3	1.6
Link to external resource (media)	Comments promoting news pieces without relating them to the preprint's content.	"http://www.businessworld.in…" (http://disq.us/p/290wdok)	3	1.6

© 2023 Carneiro CFD et al. *JAMA Network Open.*

Category	Description	Example	N	%
Access to materials	Comments about data availability without contextualizing with the preprint's content	"Is the xiFDR v2.0 software available? The Rappsilber lab link only seems to provide version 1.4.3.1" (http://disq.us/p/29iwn47)	2	1.0
Importance of science	Comments thanking authors for doing research.	"I'm a non health or research person. But I thank you and others who are quickly researching and reporting on observations. (…)" (http://disq.us/p/29j0m7h)	2	1.0
Interaction with stakeholders (policy-makers)	Comments about governmental/industry regulations related to the proposals of the study.	"Very odd FDA put this on hold" (http://disq.us/p/2bc4pil)	2	1.0
Public engagement	Comments asking for non-technical explanations of the study.	" I recently taught HS Biology but this is beyond my understanding. Is there anyone who can explain this study to me in simple terms?" (http://disq.us/p/28zady5)	2	1.0
Apology	Comment apologizing for previous criticisms.	"(…) I would like to apologize to the EV research community, the authors of this paper and specifically to (…)" (http://disq.us/p/2bpcog6)	1	0.5
Authorship	Questions whether a reference is by the same authors.	"https://www.preprints.org/m… Is this the same team?" (http://disq.us/p/295q4kb)	1	0.5
Conflict of interest	Comment about the declarations on conflicts of interest of the study.	"Note the massive conflicts of interest for these "experts." (…) Can we also get a full disclosure from Mr. Weinberger on any personal investments he may have in pharmaceutical companies or anything related to that industry." (http://disq.us/p/28y127q)	1	0.5
Duplication of comments	Comment about the duplication of a previous comment.	"This accidentally double-posted, due to editor approval taking longer than expected." (http://disq.us/p/2bou13j)	1	0.5
Interaction with stakeholders (industry)	Comment about manufacturing support for the proposal of the study.	"It would be great if you would have any real manufacturing support." (http://disq.us/p/2b60v54)	1	0.5
Research practices	Comment with questions and criticisms about research practices.	"These vaccine makers will take billions of dollars experimenting on us before any of their mistakes or lies can be pointed out." (http://disq.us/p/2fkutm5)	1	0.5

© 2023 Carneiro CFD et al. *JAMA Network Open.*

eTable 5 - Types of references cited in comments. N is the number of comments including at least one reference of each type (number of references per comment were not recorded), and percentages are relative to 284 fully assessed comments with references. Comments that included isolated links with no other content were not assessed at this stage, and are described in eTable 4. Categories are not mutually exclusive (i.e. each comment could have multiple types of references); thus, percentages don't add up to 100%. References classified as 'other' included a Wikipedia page, a Google search output, a pre-registration, a presentation, an online tool and guideline documents, plus other references of unclear type.

**Category**	**N**	**%**
Journal article	186	65.5
Preprint	61	21.5
Blog/website	43	15.1
Governmental document	10	3.5
Data	8	2.8
Manual/documentation	8	2.8
News piece	5	1.8
Social media	3	1.1
Book	2	0.7
Other	13	4.6

© 2023 Carneiro CFD et al. *JAMA Network Open.*

eTable 6 - Types of organized review efforts. 75 comments were identified as being from an organized review effort. Classifications of the type of effort were made after completion of data collection (see Methods for details). Comments that included links to external review platforms and no other content were not assessed, as they were categorized as not about the preprint and included in eTable4.

**Type of organized review effort**	**n**	**%**
Institutionally organized review	55	73.3
Lab review or journal club	7	9.5
Automated screening tool	5	6.7
Course review	4	5.3
Institutional journal club	3	4.0
Journal-requested review	1	1.3

© 2023 Carneiro CFD et al. *JAMA Network Open.*

eTable 7 - Counts (N) and examples of specific content of comments, ordered by total number in each category. The complete text of all comments and their links are available at https://osf.io/quz6f and https://osf.io/vg6at.

	Criticisms, corrections or suggestions		Compliments		Questions	
**Category**	**N**	**Example**	**N**	**Example**	**N**	**Example**
Interpretation	286	"Your conclusion is wrong. Both weather and public intervention could impact on the number of cases. (…)" (http://disq.us/p/283x9c7)	16	"(…) The evolutionary aspects of living things are what most excite me and I find the interpretation of the authors of this work, fascinating. (…)" (http://disq.us/p/27zj3aa)	129	"Don't you think that lung capacity differences are instead the key to explain the lower incidence and severity of the disease in women and children? Thank you." (http://disq.us/p/28rl8jq)
Materials and data collection	238	"(…) This is simply an artifact of BLAST search mistakenly aligning sequences between distantly related genomes. (…)" (http://disq.us/p/27wbns2)	21	"(…) Thanks for (sic) much for following best practice and using PRIME-NTD.(…)" (http://disq.us/p/2f71y2w)	166	"(…) Is it possible to control cluster size experimentally, for example by adjusting fixation conditions or sonication conditions, to optimise for detection of a specific class of interactions?" (http://disq.us/p/2cdv3h8)
Methodological design	267	"If you want to curb covid, or if you want to write off medication as being useless "for covid", start doing trials on early outpatient treatment." (http://disq.us/p/2bf75n0)	42	"(…) I am glad to see that in this pre-print controls were shown, which were not provided earlier, such as the effect of substrate on the apparent diffusion of the dye and other enzymes. (…)" (http://disq.us/p/29dfp0g)	112	"(…) is it the same subject with different samples? (…)" (http://disq.us/p/2c9v9cr)
Analysis	228	"(…) Sorry, but it's simply not true that you can calculate affinity and efficacy from those data. (…)" (http://disq.us/p/26u1dyr)	24	"(…) Good to see adjustment for 16 covs. and sens analysis. (…)" (http://disq.us/p/28h6m7q)	101	"(…) Can we be sure that all the roots were sampled or would it makes [sic] sense to include depth as an additional predictor in the random forest?" (http://disq.us/p/26xq3rf)
Additional information	146	"(…) Also, it would be useful to see a table with the match qualities, and an	1	"(…) Furthermore, we admire the interactive presentation of volume and	170	"Hi! Is there any information on how much each of those underlying health

© 2023 Carneiro CFD et al. *JAMA Network Open.*

Category	Criticisms, corrections or suggestions		Compliments		Questions	
	N	Example	N	Example	N	Example
		example chromatogram. (…)" (http://disq.us/p/26r8bbe)		surface data via interactive web tools. (…)" (http://disq.us/p/2a14b06)		conditions increases risk of severe COVID-19 disease? Thanks" (http://disq.us/p/28xx2ix)
Implications	133	"(…) Further study should test SARS-CoV-2 in CD147 positive blood cell from COVID-19 patients." (http://disq.us/p/288ebcx)	72	"(…) I belive [sic] this study will underpin ongoing work on animal functional genomics, environmental adaptation and developmental evolution." (http://disq.us/p/289c5ct)	69	"(…) Have you researched (or have plans to) how CQ/HCQ react with other diabetes drugs apart from Metformin (e.g., Glimepiride, Vildagliptin, etc.)? (…)?" (http://disq.us/p/28fpv76)
Concepts/ theoretical framework	163	"The authors built their research on the finding of (…) and the similarity between SARS-CoV-2 SARS-CoV spike protein [S not SP as mentioned by the authors]. (…)" (http://disq.us/p/27yiost)	11	"Happy to see these results contesting a genetic discontinuity. (…)" (http://disq.us/p/2h0biqf)	88	"(…) In fact, has any study conclusively proved that Sars-Cov-2 causes COVID-19?" (http://disq.us/p/2anl8mo)
Reporting	181	"Is it possible to get a better and more detailed description of Materials & Methods. We are not able to understand it the way it is described here." (http://disq.us/p/2bkvalu)	11	"(…) The authors have made sure that there is no bias in selection and reporting the evidences [sic] through use of appropriate software and methods." (http://disq.us/p/2e0tn8i)	76	"(…) could you please explain why you have not uploaded the STROBE guidelines? (…)" (http://disq.us/p/28v148t)
Previous literature	183	"(…) The preprint makes several false claims, which is unsurprising when considering that the literature cited does not extend beyond 2007!" (http://disq.us/p/2c5lp35)	5	"(…) It is cool to see a Tweet thread as a major reference in a preprint article that is receiving press attention. (…) Changes in science communication are spreading as fast as the virus. (…)" (http://disq.us/p/29ib3jh)	32	"Why is there no reference to this well-powered study by Cordi et al. (2014) (...)?" (http://disq.us/p/2bkovki)
Relevance	39	"(…) There should have been a broader base of information to permit placing symptomatology in a more useful	111	"Very nice and important finding" (http://disq.us/p/285rsao)	5	"Hi. Most of your theoretical peptides have 1-2 miscleavages. How these peptides are [sic] useful at all for SRM

© 2023 Carneiro CFD et al. *JAMA Network Open.*

Category	Criticisms, corrections or suggestions		Compliments		Questions	
	N	Example	N	Example	N	Example
		perspective. (…)” (http://disq.us/p/2cix1zx)				or PRM methods?” (http://disq.us/p/28guupl)
Readability	105	“(…) I don't think consensi as a plural of consensus exists in english (it does in Italian though!). I might be wrong. (…)” (http://disq.us/p/27qdvc5)	19	“(…) your lay summary is really nice; congrats (…)” (http://disq.us/p/28qyx9u)	19	“Hi, Can you clarify the meaning of the theta "infection" parameter in equation 1 (years 2015-2019) which multiplies the death rate? Is this a typo, or set to 1?” (http://disq.us/p/298ifrk)
Data visualization	81	“This might have already been picked up, but in Figure 4F q2, "Glomeromycotina" is included twice in the quartet! The top one should be "Mucoromycotina".” (http://disq.us/p/2ck48u3)	8	“Really interesting paper and visualization approach! (…)” (http://disq.us/p/2dtn5jq)	25	“When I look at Predicted Case Rate, I cannot find MO. It does show up on the death rate chart. Am I just missing it or is MO missing from the Case chart?” (http://disq.us/p/2co9z67)
Data sharing	52	“(…) Source code merely shows how to generate output.<br>The data should be openly available, given the supervising author's history.” (http://disq.us/p/2buytvg)	3	“(…) Moreover, all data generated in this study is publicly available for further investigation, which is critical for clinical translation in the evolving pandemic. (…)” (http://disq.us/p/28l2mo7)	39	“(…) Is it possible to inform these missing information [sic]? (…)” (http://disq.us/p/2dk3zm1)
Novelty/ Originality	25	“(…) This article is of limited significance as it simply reports similar descriptions of COVID patients made in previous literature that severe cases are characterized by lymphopenia.” (http://disq.us/p/285n7u0)	26	“A novel topic chosen for systematic review and meta-analysis have [sic] medical implication for developing countries. (…)” (http://disq.us/p/2e0vp0d)	6	“Has this type of study not already been performed in other countries before this? (…)” (http://disq.us/p/2b4gp37)
Ethics	26	“This paper has lifted many portions of texts in several places from the paper	0	-	7	“Could the authors please clarify whether the study has been commissioned by a corporate client as

© 2023 Carneiro CFD et al. *JAMA Network Open.*

Category	Criticisms, corrections or suggestions		Compliments		Questions	
	N	Example	N	Example	N	Example
		https://hal.archives-ouvert... by (…)” (http://disq.us/p/29fjbk0)				suggested (…)?” (http://disq.us/p/2fx3iu6)
Title/Abstract	22	“I am sorry to say, but the title is somewhat misleading, the authors did not show that (…). What the authors show is that (…)” (http://disq.us/p/28fmgkb)	4	“(…) I will start out with positive; the abstract was greatly laid out. I like how it is broken down to individual parts. (…)” (http://disq.us/p/2covspc)	1	“Based on the information described above and the data presented by the authors we think that the title is not valid to this work. We would be grateful if the authors can provide more information about their research that could have a positive impact on the veterinary field.” (http://disq.us/p/2dlf8k8)

© 2023 Carneiro CFD et al. *JAMA Network Open.*

eTable 8 - Analysis of comment content using the preprint as the unit of analysis. N refers to the number of preprints with at least one comment falling under the category in question. Percentages refer to the total number of preprints with at least one non-author comment about the preprint's content (810 preprints). The content categories are not mutually exclusive; thus, the sum of percentages is greater than 100%.

**Category**	**N**	**%**
Includes a criticism, correction or suggestion	554	68.4
Includes a compliment or positive appraisal	388	47.9
Includes a question	328	40.5
Includes content that was not classified above	65	8.0
Includes references	252	31.1
Includes a summary description	103	12.7
Explicitly questions the conclusion of the article	89	11.0
Provides new data	12	1.5
Provides new analyses	4	0.5
Provides both new data and analyses	4	0.5
Presented clearly enough for understanding	799	98.6
From an organized review effort	72	8.9
Offensive (to the authors)	2	0.25

© 2023 Carneiro CFD et al. *JAMA Network Open.*

eTable 9 – Analysis of selected subsets. The first two columns present the same data as Table 1, while all others represent subsets of this data. Percentages refer to the total number of comments in each category. The content categories are not mutually exclusive; thus, the sum of percentages is greater than 100%.

**Category**	**Complete dataset**		**From organized review efforts (n=75)**		**Questioning conclusions (n=98)**		**With a response from authors (n=200)**		**With a response from nonauthors (n=91)**	
	**N**	**%**	**N**	**%**	**N**	**%**	**N**	**%**	**N**	**%**
Includes a criticism, correction or suggestion	694	**61.7**	74	**98.7**	93	**94.9**	125	**61.6**	56	**61.5**
Includes a compliment or positive appraisal	428	**38.0**	39	**52.0**	5	**5.1**	79	**38.9**	22	**24.2**
Includes a question	393	**34.9**	9	**12.0**	17	**17.3**	87	**42.9**	40	**44.0**
Includes content that was not classified above	69	**6.1**	4	**5.3**	0	**0.0**	4	**2.0**	6	**6.6**
Includes references	284	**25.2**	33	**44.0**	31	**31.6**	55	**27.1**	16	**17.6**
Includes a summary description	110	**9.8**	66	**88.0**	7	**7.1**	11	**5.4**	6	**6.6**
Explicitly questions a conclusion of the article	98	**8.7**	1	**1.3**	-	**-**	11	**5.4**	16	**17.6**
Provides new data	12	**1.1**	0	**0**	7	**7.1**	1	**0.5**	4	**4.4**
Provides new analyses	5	**0.4**	0	**0**	1	**1.0**	2	**1.0**	1	**1.1**
Provides both new data and analyses	4	**0.4**	0	**0**	2	**2.0**	1	**0.5**	0	**0.0**
Presented clearly enough for understanding	1109	**98.6**	75	**100.0**	97	**99.0**	200	**98.5**	89	**97.8**
From an organized review effort	75	**6.7**	-	**-**	1	**1.0**	5	**2.5**	4	**4.4**
Offensive (to the authors)	2	**0.2**	0	**0**	0	**0**	0	**0**	0	**0**

© 2023 Carneiro CFD et al. *JAMA Network Open.*

eTable 10 – Exploratory associations with categorical preprint features. Each cell includes the % of comments in each combination of categories, in relation to the total number of applicable comments: 1,482 for “is from one of the authors”, 1,317 for “is about the content” and 1,125 for all other categories. Sample sizes presented in the table represent number of comments in each category. Logistic regressions were performed where each content category was taken as the response variable (using “No” as the reference), presenting p-values from analysis of deviance tests. The content categories are not mutually exclusive; thus, the sum of percentages is greater than 100%.

		Questions conclusions of the article	Is from one of the authors	Is from an organized effort	Is about the content	Includes references	Includes questions	Includes other specific content	Includes criticisms, corrections or suggestions	Includes compliments or positive appraisals	Includes a summary description
**COVID-related**	**Yes (n=1019)**	10.8%	8.4%	7.6%	82.6%	20.6%	35.5%	7.3%	62.1%	29.4%	9.9%
	**No (n=459)**	4.3%	17.2%	4.6%	92.1%	35.4%	33.4%	3.4%	61.1%	56.6%	9.7%
	**p-value**	1.5×10^{-4}	1.6×10^{-6}	0.048	3.6×10^{-6}	2.1×10^{-7}	0.491	0.009	0.753	7.1×10^{-18}	0.941
**Platforms**	**medRxiv (n=782)**	10.4%	11.4%	6.3%	86.1%	19.4%	35.7%	6.9%	63.1%	30.5%	10.2%
	**bioRxiv (n=700)**	6.8%	10.9%	6.8%	84.6%	31.8%	34.1%	5.3%	60.0%	46.6%	9.3%
	**p-value**	0.032	0.749	0.848	0.432	1.8×10^{-6}	0.577	0.273	0.284	2.7×10^{-8}	0.599
**Areas, bioRxiv**	**Genetics (n=32)**	7.7%	9.4%	0%	89.6%	23.1%	46.1%	0%	57.7%	34.6%	0%
	**Mol. Biol. (n=26)**	0%	19.2%	5.6%	85.7%	27.8%	50.0%	0%	50.0%	72.2%	11.1%
	**Genomics (n=32)**	10.7%	6.2%	7.1%	93.3%	39.3%	21.4%	3.6%	78.6%	42.9%	7.1%
	**Biochem. (n=36)**	5.0%	36.1%	10.0%	87.0%	45.0%	35.0%	5.0%	55.0%	35.0%	10.0%
	**Evol. Biol. (n=39)**	7.1%	17.9%	0%	87.5%	32.1%	32.1%	7.1%	57.1%	42.6%	7.1%
	**Cell Biol. (n=31)**	3.8%	0%	11.5%	83.9%	26.9%	7.7%	3.8%	69.2%	57.7%	19.2%
	**Immunol. (n=58)**	8.1%	5.2%	5.4%	67.3%	29.7%	48.6%	5.4%	56.8%	48.6%	5.4%
	**Neurosci. (n=57)**	0%	15.8%	11.4%	91.7%	45.4%	29.5%	4.5%	68.2%	59.1%	18.2%

© 2023 Carneiro CFD et al. *JAMA Network Open.*

		Questions conclusions of the article	Is from one of the authors	Is from an organized effort	Is about the content	Includes references	Includes questions	Includes other specific content	Includes criticisms, corrections or suggestions	Includes compliments or positive appraisals	Includes a summary description
	**Bioinform. (n=50)**	4.6%	4.0%	4.6%	89.6%	30.2%	30.2%	9.3%	65.1%	39.5%	4.6%
	**Microbiol. (n=178)**	9.6%	5.1%	11.1%	79.9%	27.4%	33.3%	7.4%	58.5%	32.6%	11.1%
	**p-value**	0.222	$5.1x10^{-6}$	0.161	0.029	0.482	0.019	0.594	0.527	0.016	0.136%
**Areas, medRxiv**	**Intens. Care (n=6)**	0%	16.7%	20.0%	100%	40.0%	40.0%	0%	60.0%	80.0%	20.0%
	**Health Pol. (n=7)**	20.0%	28.6%	0%	100%	0%	40.0%	20.0%	60.0%	0%	0%
	**Oc. Health (n=8)**	0%	0%	0%	87.5%	14.3%	71.4%	14.3%	57.1%	0%	0%
	**Resp. Med. (n=19)**	0%	5.3%	20.0%	83.3%	13.3%	26.7%	0%	73.3%	46.7%	26.7%
	**Health Inf. (n=13)**	11.1%	23.1%	0%	90.0%	11.1%	22.2%	0%	88.9%	22.2%	0%
	**Gen. Med. (n=17)**	12.5%	0%	6.2%	94.1%	12.5%	62.5%	6.2%	43.7%	37.5%	6.2%
	**Al. Immun. (n=22)**	0%	0%	26.3%	86.4%	21.0%	31.6%	10.5%	63.2%	47.4%	26.3%
	**Pub. Health (n=46)**	8.3%	17.4%	0%	94.7%	13.9%	33.3%	11.1%	47.2%	47.2%	8.3%
	**Epidemiol. (n=191)**	13.5%	14.1%	2.0%	90.8%	22.1%	32.9%	9.4%	63.1%	24.2%	6.7%
	**Infect. Dis. (n=362)**	11.7%	6.3%	9.2%	80.5%	20.1%	37.0%	4.8%	67.0%	24.9%	12.8%
	**p-value**	0.191	0.002	$9.0x10^{-4}$	0.030	0.705	0.294	0.336	0.226	$7.8x10^{-4}$	0.061

© 2023 Carneiro CFD et al. *JAMA Network Open.*

eTable 11 – Exploratory associations with continuous preprint features. Means and standard deviations (s.d.) of each variable in each category are presented. Sample sizes presented in the table represent number of comments in each category. Logistic regressions were performed where each content category was taken as the response variable (using “No” as the reference), presenting p-values from analysis of deviance tests. Number of words refer to the length of the comments, all other variables are from the preprints.

		Questions conclusions of the article		Is from one of the authors		Is from an organized effort		Is about the content		Includes references		Includes questions		Includes other specific content		Includes criticisms, corrections or suggestions		Includes compliments or positive appraisals		Includes a summary description	
		Yes	No	Yes	No	Yes	No	Yes	No	Yes	No	Yes	No	Yes	No	Yes	No	Yes	No	Yes	No
	**n**	98	1026	165	1317	75	1050	1125	192	284	841	393	732	69	1056	694	431	428	697	110	1015
**Number of words**	**mean**	**178**	**122**	**36**	**114**	**632**	**93**	**127**	**40**	**241**	**89**	**119**	**131**	**108**	**128**	**173**	**52**	**166**	**103**	**539**	**84**
	**s.d.**	234	264	56	246	652	164	262	80	457	127	295	242	185	266	322	57	327	209	601	139
	**p-value**	0.081		2.1×10^{-15}		2.5×10^{-33}		2.7×10^{-17}		2.9×10^{-17}		0.464		0.515		1.2×10^{-35}		1.1×10^{-4}		1.6×10^{-46}	
**Altmetric score**	**mean**	**642**	**545**	**90**	**568**	**426**	**561**	**553**	**659**	**348**	**622**	**593**	**531**	**753**	**539**	**540**	**574**	**395**	**649**	**382**	**571**
	**s.d.**	1124	1196	273	1209	1109	1204	1198	1270	842	1290	1282	1151	1542	1172	1116	1321	1071	1261	1071	1210
	**p-value**	0.456		2.2×10^{-14}		0.319		0.272		2.7×10^{-4}		0.409		0.181		0.643		3.1×10^{-4}		0.089	
**Citations**	**mean**	**99**	**191**	**24**	**177**	**189**	**182**	**183**	**146**	**71**	**220**	**171**	**189**	**138**	**185**	**191**	**168**	**117**	**222**	**139**	**187**
	**s.d.**	376	1129	74	1153	442	1215	1179	988	306	1350	1031	1252	456	1211	1190	1162	947	1300	379	1235
	**p-value**	0.389		1.1×10^{-5}		0.958		0.669		0.018		0.804		0.729		0.750		0.120		0.660	
**Impact factor**	**mean**	**9.1**	**14.1**	**9.5**	**14.8**	**17.8**	**13.4**	**13.8**	**20.8**	**10.8**	**14.7**	**12.8**	**14.4**	**15.0**	**13.7**	**13.8**	**13.8**	**13.5**	**14.0**	**16.5**	**13.5**
	**s.d.**	12.8	19.1	10.7	19.0	17.2	18.9	18.8	19.6	12.8	20.2	17.4	19.6	16.9	18.9	18.8	18.9	17.6	19.6	16.6	19.1
	**p-value**	0.232		0.022		0.318		0.034		0.111		0.488		0.813		0.987		0.834		0.427	
**Number of comments**	**mean**	**3.5**	**3.4**	**1.8**	**3.4**	**2.8**	**3.4**	**3.4**	**3.5**	**2.8**	**3.6**	**3.7**	**3.2**	**3.8**	**3.3**	**3.2**	**3.7**	**2.8**	**3.7**	**2.8**	**3.4**
	**s.d.**	3.9	3.7	1.5	3.8	3.6	3.8	3.8	3.8	3.0	4.0	4.1	3.6	4.6	3.7	3.5	4.1	3.1	4.1	3.4	3.8
	**p-value**	0.658		2.3×10^{-11}		0.188		0.754		0.002		0.065		0.308		0.019		9.3×10^{-5}		0.068	

© 2023 Carneiro CFD et al. *JAMA Network Open.*

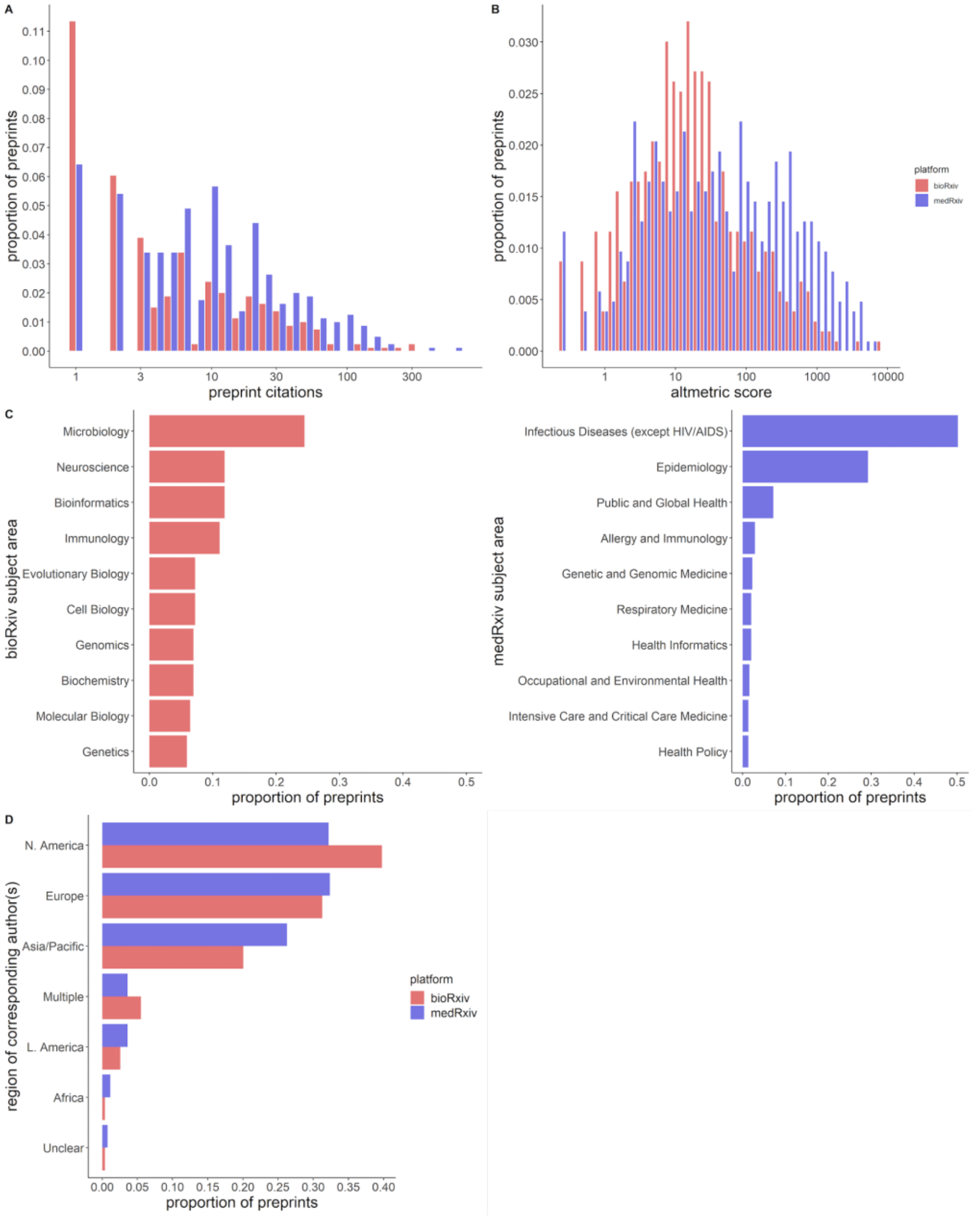

eFigure 1 – Preprint features. (A) Distribution of number of citations to the preprint (obtained via Crossref on Oct. 28th 2021). Median (interquartile range) score is 1 (0 – 6) for bioRxiv preprints and 6 (2 – 19) for medRxiv. Overall median (interquartile range) is 3 (1 – 12). 240 preprints (23.2%) did not receive any citations. **(B)** Distribution of Altmetric scores. Median (interquartile range) score for bioRxiv preprints is 14.8 (4.6 – 48.2) and 37.0 (6.2 – 262.4) for medRxiv. Overall median (interquartile range) is 21.2 (5.5 – 119.4). Scores were unavailable for 3 preprints. **(C)** Areas of research. Given the large number of areas with very few preprints in our sample, we only show the 10 most prevalent areas for bioRxiv (*left*) and for medRxiv (*right*). **(D)** Region of origin of the corresponding authors. 'Multiple' combines cases where the corresponding author had affiliations in more than one region or where multiple corresponding authors had affiliations in different regions. Region of origin could not be identified for 18 preprints.

© 2023 Carneiro CFD et al. *JAMA Network Open.*

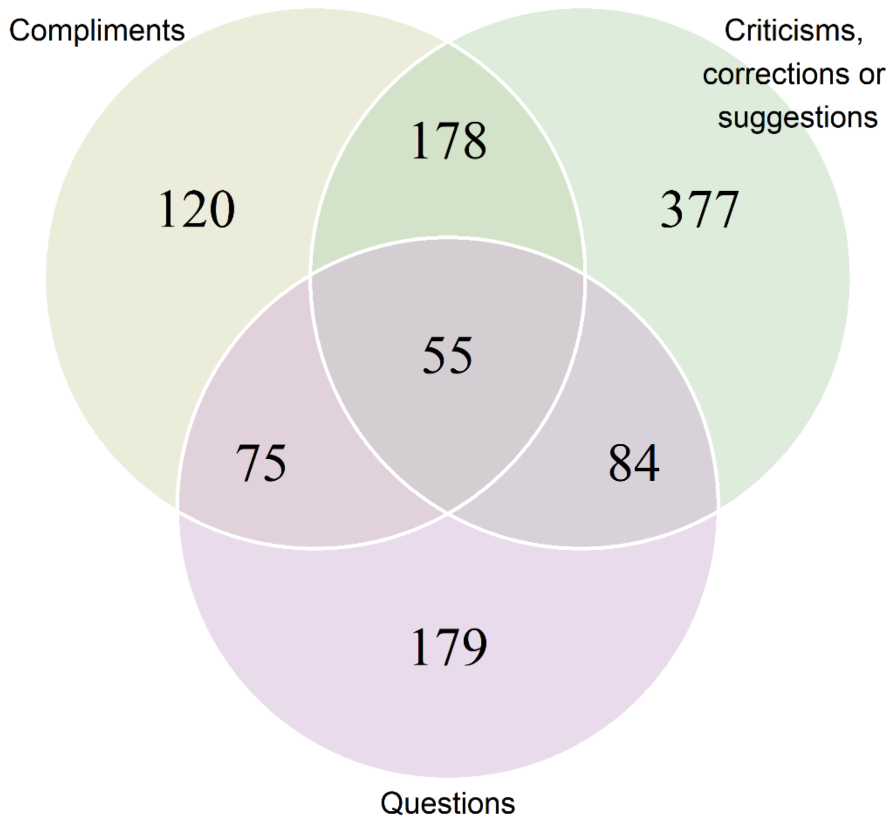

eFigure 2 - Venn diagram representing the overlap between the main content categories within comments. Areas of overlap between circles are not proportional to the number of comments in each of them.

© 2023 Carneiro CFD et al. *JAMA Network Open.*

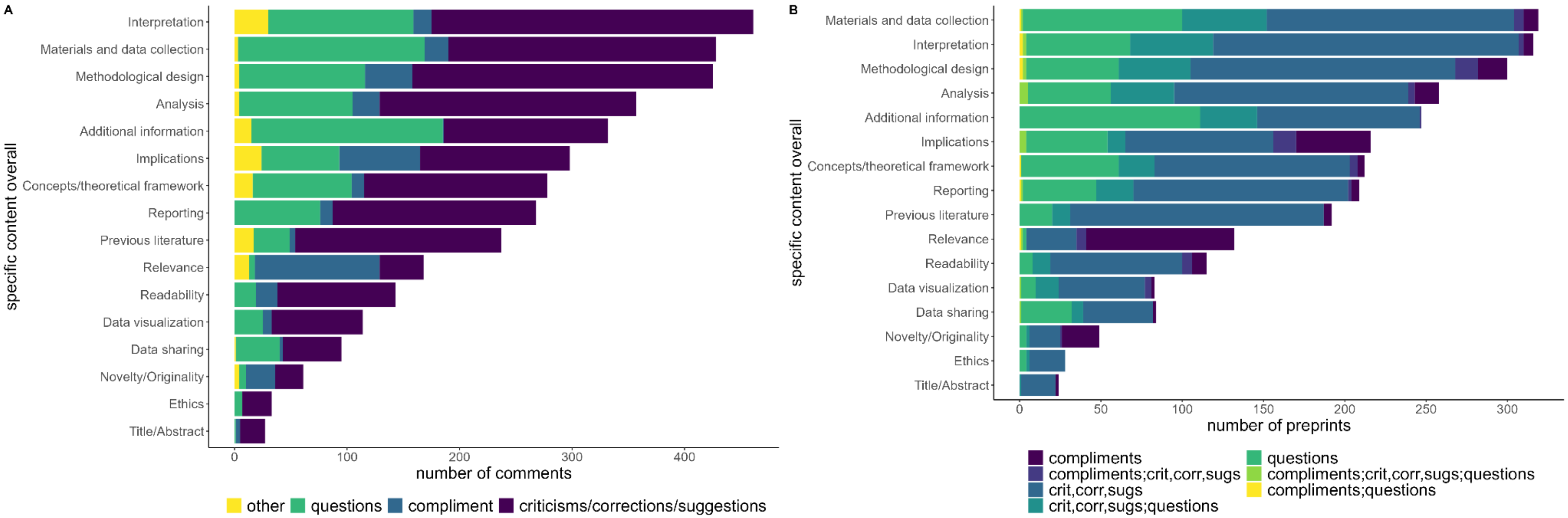

eFigure 3 – Specific content in all main categories, including criticisms/corrections/suggestions, compliments, questions and comments not classified in any of these categories (other). (A) Number of comments including each specific subcategory. (B) Number of preprints with at least one comment including each specific subcategory.

© 2023 Carneiro CFD et al. *JAMA Network Open.*

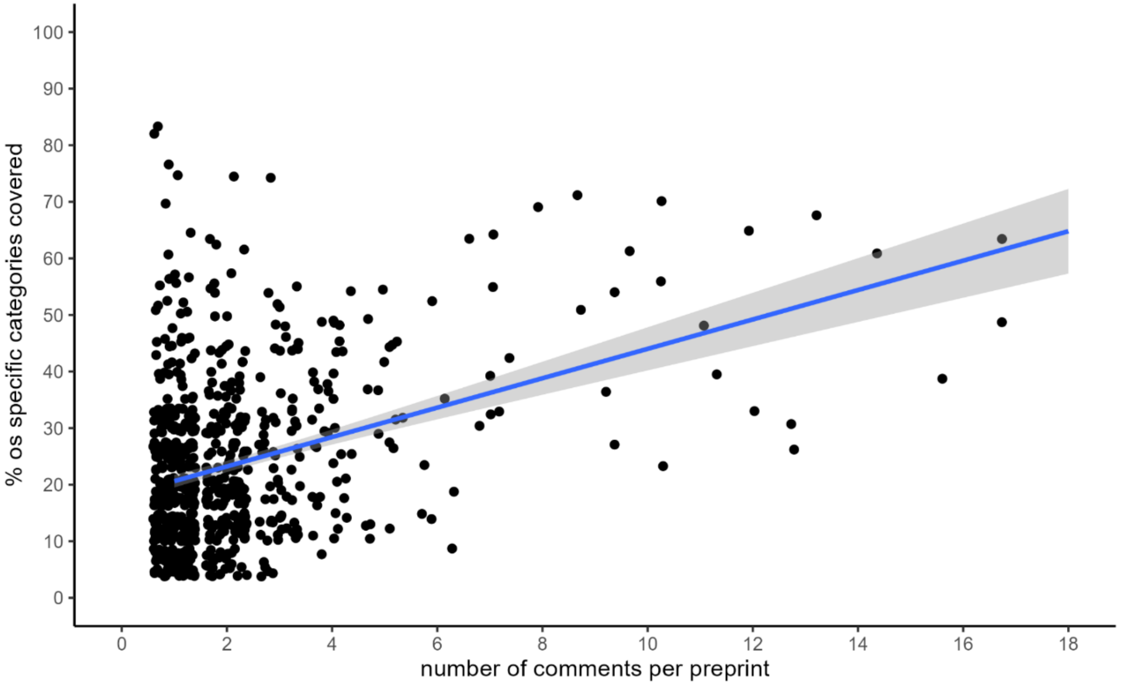

eFigure 4 - Correlation between the number of comments per preprint and the percentage of specific categories addressed by the aggregate of all comments. Specific content categories are: Novelty/Originality, Relevance, Readability, Previous literature, Concepts/Theoretical framework, Title/Abstract, Methodological design, Materials and data collection, Ethics, Data visualization, Analysis, Interpretation, Implications, Data sharing, Reporting, Additional information. The percentage presented in the figure is among all comments, regardless of them have been classified previously as a criticism, correction or suggestion, as a compliment or as a question. Pearson's r = 0.37, p = 2.2×10^{-16}, n = 738 preprints. Regression line is shown in blue, with the gray area representing its 95% confidence interval.

© 2023 Carneiro CFD et al. *JAMA Network Open.*
