# Supplementary material for: Characterization of Comments About bioRxiv and medRxiv Preprints
Source: JAMA Netw Open. 2023 Aug 30;6(8):e2331410. doi: 10.1001/jamanetworkopen.2023.31410 (PMC10469270; doi:10.1001/jamanetworkopen.2023.31410)
Supplement: Supplement 2. — Data Sharing Statement [file jamanetwopen-e2331410-s002.pdf]

Data Sharing Statement

Carneiro. Characterization of Comments About bioRxiv and medRxiv Preprints. *JAMA Netw Open*. Published August 30, 2023. doi:10.1001/jamanetworkopen.2023.31410

Data
Data available: Yes
Data types: Data (not involving human participants)
How to access data: https://osf.io/k9e8c/
When available: beginning date: 09-26-2022

Supporting Documents
Document types: Statistical/analytic code
How to access documents: https://osf.io/k9e8c/
When available: beginning date: 09-26-2022

Additional Information
Who can access the data: Access to the data will not be restricted, anyone with the link can access.
Types of analyses: All analyses presented in the main manuscript, as well as supplemental material, are available, including the scripts for generation of the figures and tables.
Mechanisms of data availability: Access to the data will not be restricted, anyone with the link can access.
Any additional restrictions: All content is shared under a CC-BY license.
